# Anisotropic attosecond charge carrier dynamics and layer decoupling in quasi-2D layered SnS_2_

**DOI:** 10.1038/s41467-017-01522-3

**Published:** 2017-11-08

**Authors:** Calley N. Eads, Dmytro Bandak, Mahesh R. Neupane, Dennis Nordlund, Oliver L. A. Monti

**Affiliations:** 1 Department of Chemistry and Biochemistry, University of Arizona, 1306 East University Boulevard, Tucson, AZ 85721 USA; 2 Sensors and Electron Devices Directorate, US Army Research Laboratory, Adelphi, MD 20783 USA; 3 SLAC National Accelerator Laboratory, Stanford Synchrotron Radiation Lightsource, 2575 Sand Hill Road, MS 99, Menlo Park, CA 94025 USA; 4 Department of Physics, University of Arizona, 1118 East Fourth Street, Tucson, AZ 85721 USA

## Abstract

Strong quantum confinement effects lead to striking new physics in two-dimensional materials such as graphene or transition metal dichalcogenides. While spectroscopic fingerprints of such quantum confinement have been demonstrated widely, the consequences for carrier dynamics are at present less clear, particularly on ultrafast timescales. This is important for tailoring, probing, and understanding spin and electron dynamics in layered and two-dimensional materials even in cases where the desired bandgap engineering has been achieved. Here we show by means of core–hole clock spectroscopy that SnS_2_ exhibits spin-dependent attosecond charge delocalization times (*τ*_deloc_) for carriers confined within a layer, *τ*_deloc_ < 400 as, whereas interlayer charge delocalization is dynamically quenched in excess of a factor of 10, *τ*_deloc_ > 2.7 fs. These layer decoupling dynamics are a direct consequence of strongly anisotropic screening established within attoseconds, and demonstrate that important two-dimensional characteristics are also present in bulk crystals of van der Waals-layered materials, at least on ultrafast timescales.

## Abstract (web summary)

Owing to their layered nature, transition metal dichalcogenides possess an anisotropic electronic structure whose impact on carrier dynamics is not fully known. Here, the authors use X-ray spectroscopy to unveil the electronic coupling and attosecond dynamics in SnS_2_, a prototypical van der Waals layered crystal.

## Introduction

In recent years, the family of two-dimensional (2D)-layered transition metal dichalcogenides (TMDs) has gained significant interest due to their unique layer-dependent electronic properties, including layer-dependent band structures^1^, transition from indirect-to-direct bandgap^2,3^, and the ability to support substantial spin^4,5^ and valley^6–8^ polarization. Consequently, TMDs show promise as highly efficient field-effect transistors^9,10^, photovoltaics^11^ and spintronic devices^12^, and offer new avenues toward quantum computing. This promise hinges on a detailed understanding of the fundamental physics at play in TMDs, with particular emphasis on the spatially highly anisotropic electronic properties and the resulting consequences of quantum confinement in 2D. Indeed, exquisite band structure measurements using state-of-the-art angle-resolved photoemission (ARPES) have elucidated the electronic structure of TMDs^13–15^ and opened avenues toward tailoring bandgap and electronic properties, e.g., with different interlayer twist angles^16^. Despite this emerging body of ARPES work and while the electronic structure and excitations in TMDs have been investigated widely^14,17,18^, the extent to which the anisotropic electronic structure of layered materials confers 2D character already in the bulk crystals is not yet fully understood. If bulk crystals already exhibit essential aspects of 2D materials, simplification of device fabrication protocols may be anticipated, broadening their use in novel electronic devices. A number of studies have indeed suggested that layers in bulk TMDs, such as ReS_2_^19^, WSe_2_^20^, MoS_2_^21^, or in graphite^22^, are sufficiently electronically decoupled to present as 2D materials, e.g., in terms of spin polarization. The observation of anisotropic screening and carrier dynamics in the time-domain would constitute a direct probe of layer decoupling in bulk crystals, but such studies are at present missing due to the extremely short timescales involved and the difficulty of spectroscopically resolving the anisotropic dynamical processes.

Here we address this open question by investigating the ultrafast carrier dynamics in the layered semiconductor SnS_2_^23–25^. Using core–hole clock spectroscopy^26^, we observe spin-dependent anisotropic charge transfer on attosecond timescales in quasi-2D bulk SnS_2_, and show that already in bulk crystals individual layers are indeed strongly decoupled and essentially 2D. Past ultrafast spectroscopic studies have primarily focused on excitonic carrier-relaxation dynamics in bulk TMDs^27,28^ with timescales of <100 fs in WSe_2_^29^, MoS_2_^30^, and SnS_2_^31^. These measurements lack the atomic-scale specificity and time resolution to fully probe the anisotropic-correlated electron dynamics. Our study fills this need by investigating the carrier delocalization dynamics on attosecond timescales^32^: We show for the first time that van der Waals layering leads to decoupling of individual 2D layers observed dynamically on sub-fs to few-fs timescales. We demonstrate that such dynamic layer decoupling is the origin of the predominantly 2D nature and strongly anisotropic electronic properties in bulk SnS_2_, and discuss the broader applicability to spin and electron dynamics in layered and 2D materials.

## Results

### Mapping of the conduction band of SnS_2_

**Fig. 1:**
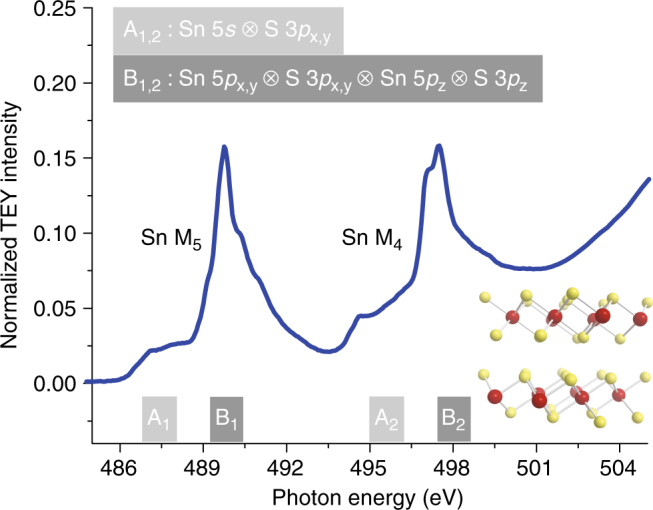
Conduction band of SnS_2_. X-ray absorption spectra of SnS_2_ on Sn M_4,5_ edges measured in total electron yield (TEY) mode, with calculated principal orbital composition indicated. All features stem from transitions in the bulk crystal (see Supplementary Note 2 for details). Inset: Layered structure of bulk SnS_2_ where red and gold spheres represent Sn and S atoms, respectively

As will be discussed in more detail below, our experimental approach is based on resonant photoemission to probe the evolution of excited states on the timescale of a core–hole decay with sub-fs time resolution. In order to properly interpret the resonant photoemission results with respect to charge-carrier dynamics, we first need to understand the character of the excited electronic states reached in the initial X-ray absorption (XA) process, in particular the excitation of electrons into the SnS_2_ conduction band. In this study, we excite from Sn 3*d* levels (M-edge) to the SnS_2_ conduction band so as to access different orbital and spin characters in the conduction band. Figure 1 shows the XA spectrum at the M-edge of SnS_2_, separated into Sn 3*d*_3/2_ (Sn M_4_) and Sn 3*d*_5/2_ (Sn M_5_) components as a result of spin–orbit coupling. Each spin–orbit component is further split into at least two distinct features, labeled A and B. Comparison to high-level electronic structure calculations^18^ allows us to assign these features to excitations of different orbital components of the conduction band (see Supplementary Note 1). The weaker features, A_1_ and A_2_, constitute excitations to regions near the conduction band minimum, composed predominately of strongly hybridized Sn 5*s* and S 3*p*_x,y_ (in-plane) levels. Since dipole selection rules dictate ${\rm{\Delta }}l = \pm 1$ (with core electron orbital angular momentum *l*), the transition into Sn 5*s* is formally forbidden. However, we still observe a weak transition due to hybridization of Sn 5*s* with S 3*p*_x,y_, resulting in orbitals/bands with dipole-allowed *p*-character. This transition represents excitation of primarily in-plane orbitals of both Sn and S character within a 2D SnS_2_ sheet. In contrast, the strong XA features, B_1_ and B_2_, constitute dipole-allowed excitations to deeper-lying levels in the conduction band, and they are composed primarily of weakly hybridized Sn 5*p*_x,y_, Sn 5*p*_z_, S 3*p*_z_, and S 3*p*_x,y_ levels. Some of these excitations access therefore out-of-plane orbitals ultimately responsible for coupling between adjacent 2D SnS_2_ sheets. The selective excitation in the XA process into these two groups of electronic levels provides the basis for probing the anisotropy of carrier dynamics in the van der Waals-layered material SnS_2_. Note that surface defects do not contribute to the observed spectral features in Fig. 1 (see Supplementary Note 2 and Supplementary Fig. 1 for details).

### Strong resonant Auger enhancement channel on Sn edge

**Fig. 2:**
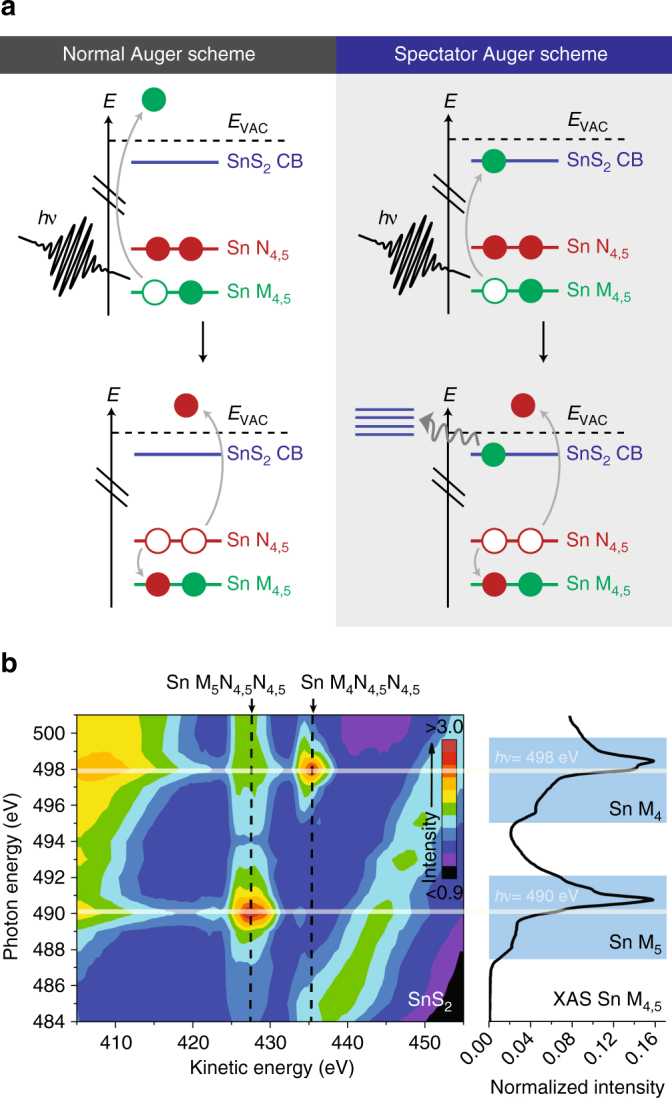
Resonant Auger scheme and intensity map. **a** Energy-level schemes of normal Auger (direct), and of spectator Auger (resonant) processes of Sn M_4,5_N_4,5_N_4,5_ when on resonance with the conduction band of SnS_2_. **b** Resonant photoemission contour plot of Sn M_4,5_N_4,5_N_4,5_ compared to TEY-XAS of Sn M_4,5_. Also shown are two lines (white, horizontal) for spectral cuts at constant photon energies 490 eV and 498 eV in Fig. 3

We access the site- and element-specific ultrafast carrier dynamics in SnS_2_ by monitoring competing decay channels following absorption of an X-ray photon from the Sn 3*d* levels into the conduction band region. Core-excited SnS_2_ decays on ultrafast timescales by Auger-like processes, which are observable with resonant photoemission spectroscopy (also referred to as resonant Auger spectroscopy)^26,33^. Figure 2 illustrates the principle: In a normal Auger process (Fig. 2a, left panel), a core-excited (core-ionized) state is initially created by direct photoemission, followed by decay of the core hole and emission of a second electron, and resulting in a two-hole final state. In contrast, in a spectator Auger process (Fig. 2a, right panel), resonant excitation of a core electron to a bound-excited state enables Auger-like core–hole decay during which the excited core electron acts as a spectator in the decay process. In this case, the resulting final state is a two-hole, one-electron final state. The corresponding Auger feature is thus resonantly shifted resulting in higher kinetic energy for the Auger electron feature: The spectator electron provides additional screening for the two-hole, one-electron final state, unless the excited spectator electron delocalizes on a timescale shorter than the core–hole lifetime of a few fs. In the latter case, the final state is a two-hole, zero-electron final state, and the Auger electron feature is identical to the normal Auger decay case. The competitive kinetics of electron delocalization and the known core–hole decay manifest themselves directly in the relative intensities of the resonantly shifted Auger (spectator Auger) and normal Auger features, and we use this approach to investigate the ultrafast electron dynamics in SnS_2_.

The resonant photoemission contour plot of Auger emission intensity as a function of photon excitation energy and photoelectron kinetic energy is shown in Fig. 2b. The total electron yield (TEY) mode absorption spectrum is obtained by integrating across all kinetic energies and shown alongside the vertical axis. Note that Auger features appear at constant values on a kinetic energy scale, indicated by vertical lines, whereas direct photoemission features disperse with photon energy, as expected for features with constant binding energy.

The intensity map shows a strong resonant enhancement of the Sn M_4,5_N_4,5_N_4,5_ Auger features when the X-ray photon energy is scanned across the Sn M_5_ and M_4_ edges (dashed vertical lines). Whereas the total enhancement of all decay channels is proportional to the combined absorption cross section, the distribution between the different channels is directly related to the electronic lifetimes in the conduction band of SnS_2_ as discussed above. We are thus able to obtain the electron dynamics from comparing the relative amplitudes of the normal and spectator Auger contributions to the Sn M_4,5_N_4,5_N_4,5_ features in Fig. 2b. Both the normal and spectator Auger feature envelopes are analyzed in the context of atomic spectroscopy considerations of electron correlation and accessible two-hole final states (see Supplementary Notes 3 and 4 for details and Supplementary Fig. 2 for the normal Auger fit). Briefly, the normal Auger features for both Sn M_4,5_N_4,5_N_4,5_ transitions correspond to a final state with *d* ^8^ atomic configuration (electronic configurations ^1^ *S*, ^1^ *G*, ^3^ *P*, ^1^ *D*, and ^3^ *F*). We fit transitions to each of these final states by incorporating state-dependent Coulombic repulsion and a screening term in the two-hole final states, giving excellent agreement with theory^34^ (see Supplementary Table 1 for quantitative results). The resulting fit parameters for the normal Auger spectra were then also used to capture the spectator Auger features after adding a spectator shift to account for the increased screening by the extra electron located in the conduction band of SnS_2_ in resonant Auger spectroscopy.

**Fig. 3:**
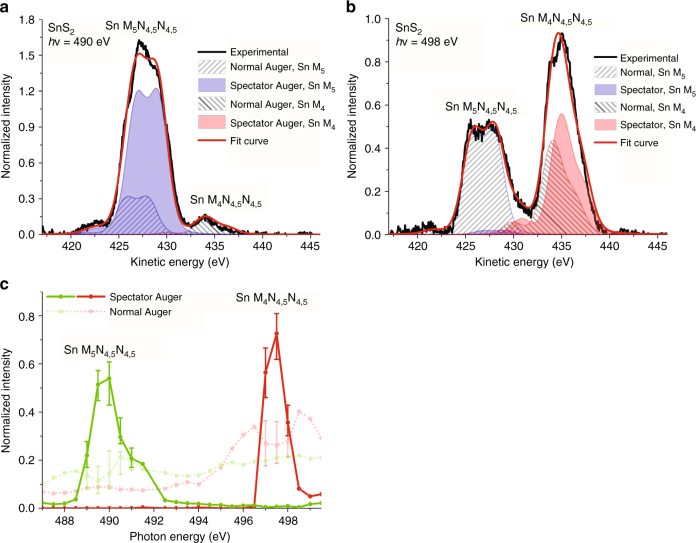
Resonant photoemission measurements at selected photon energies. Photon energy slices of the resonant photoemission spectroscopy (RPES) contour plot resonantly excited **a** on Sn M_5_N_4,5_N_4,5_ at a photon energy of 490 eV and **b** on Sn M_4_N_4,5_N_4,5_ at a photon energy of 498 eV, and displaying component fits of normal (dashed, gray) and spectator (solid) Auger features. **c** Integrated intensities of normal and spectator Auger components in the RPES contour plot for both Sn M_4_N_4,5_N_4,5_ and Sn M_5_N_4,5_N_4,5_ transitions. The error bars represent uncertainties from the fit of the spectator Auger peak areas ($\Delta {\chi ^2} \le \pm 10\%$)

Two representative decompositions of the Auger spectra are shown in Fig. 3a, b that are cut along the horizontal white guide lines in Fig. 2b, at photon energies of 490 eV (Sn M_5_ resonance) and 498 eV (Sn M_4_ resonance). At *hν* = 490 eV, the Auger spectrum is dominated by a resonantly shifted spectator Sn M_5_N_4,5_N_4,5_ Auger contribution (spectator shift of 1.1 eV), with a minor normal Sn M_5_N_4,5_N_4,5_ Auger component. There is also a small but visible excitation of normal Sn M_4_N_4,5_N_4,5_. Notably, the intensity of the spectator Auger feature is approximately four times larger than that of the normal Auger feature, indicating a long-lived excited electron that screens the core–hole decay. Similarly, at *hν* = 498 eV, the Sn M_4_N_4,5_N_4,5_ feature also has a dominant resonant Auger feature (spectator shift of 0.9 eV), but with a more significant contribution from normal Auger processes. This spectral decomposition analysis is repeated across the complete resonant photoemission map and yields the evolution of the integrated spectator and normal Auger feature intensities, as shown in Fig. 3c. The analysis shows strong resonant enhancement upon excitation of the conduction band region, and suggests the existence of long-lived carriers in some regions of the conduction band. We next use the relative magnitudes of both the normal and spectator Auger intensities to uncover the charge-carrier dynamics in SnS_2_.

### Charge delocalization within SnS_2_ sheets and localization between SnS_2_ sheets

We investigate the ultrafast charge-carrier dynamics using the core–hole lifetime as an internal clock of the excited state dynamics^26,33^. The natural lifetime of both Sn 3*d*_3/2_ and Sn 3*d*_5/2_ core–holes is identical, as predicted by theory, and verified experimentally to be *τ*_c − h_ = 1/*k*_c − h_ = 1.69 fs (refs. ^35–37^). The influence of Coster–Kronig decay channels associated with spin–orbit component-dependent lifetimes effects is not present in Sn on this edge. The experimentally accessible charge delocalization timescale is determined from the accuracy with which RPES intensities can be determined, requiring that intensities be no more than one order of magnitude apart to still enable determination of delocalization times, and hence 0.1*τ*_c − h_ ≤ *τ*_deloc_ ≤ 10*τ*_c − h_. As a result, we expect to observe competing processes on a timescale of about 170 as to 17 fs for both spin–orbit components. Under the assumption that core holes are strongly localized to an atomic site, charge transfer or delocalization of an excited electron away from the excited core diminishes the resonant contributions to the Auger spectrum in the spectator channel, as detailed above and in Fig. 2. The charge delocalization rate (*k*_deloc_ = 1/*τ*_deloc_) of the core-excited electron can thus be calculated relative to the competing intrinsic core–hole decay rate (*k*_c − h_), assuming first-order rate laws for the Auger decay^26^:

1 $${k_{{\rm{deloc}}}} = {k_{{\rm{c}} - {\rm{h}}}} \cdot \frac{{{I_{{\rm{NA}}}}}}{{{I_{{\rm{SA}}}}}}$$

where *I*_SA_ and *I*_NA_ are the intensities of the spectator and normal Auger components, respectively. The *I*_SA_/*I*_NA_ fraction varies over the investigated photon energy window of 487–499 eV, encompassing both Sn M_4,5_ absorption edges and corresponding to a photon energy-dependent *k*_deloc_. A small *I*_SA_ fraction reflects fast-charge delocalization (high *k*_deloc_), while a high fraction of *I*_SA_ represents localization of the conduction band electron near the core-excited atomic site (small *k*_deloc_). Remarkably, the energy-resolved electron dynamics vary greatly across the conduction band and even among the spin–orbit components, providing an opportunity to probe the anisotropic and spin-dependent carrier dynamics in this layered van der Waals material.

**Fig. 4:**
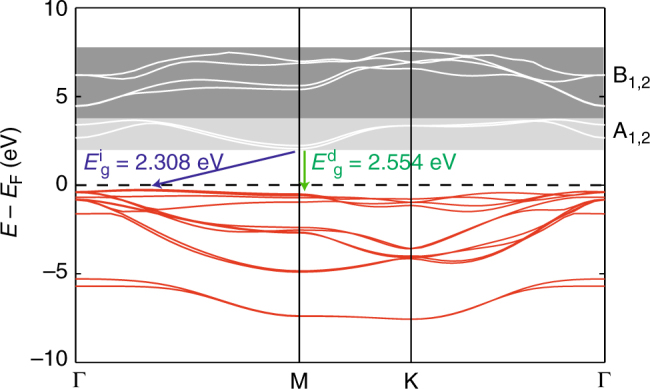
SnS_2_ band structure. Electronic band structure of bulk SnS_2_ with calculated indirect ($E_{\rm{g}}^{\rm{i}}$) and direct ($E_{\rm{g}}^{\rm{d}}$) bandgaps of 2.554 and 2.308 eV (see “Methods” for computational details). A_1,2_ and B_1,2_ indicate the regions accessed by X-ray absorption

To understand the origin of the energy-dependent dynamics, we turn to electronic structure calculations. Bulk SnS_2_ is an indirect bandgap semiconductor with a flat valence band and a maximum between the high-symmetry points Γ and M, whereas the conduction band minima are at Γ and M (see Fig. 4). The RPES results are independent of electron momentum, and we concentrate on these two representative high-symmetry points, Γ and M, to understand the energy-dependent electron dynamics. This is further justified by the relatively minor dispersion in both the valence and conduction bands, and the fact that all our conclusions are also valid at K (see Supplementary Note 5). The full band structure, including Γ to Α, is shown in the Supplementary Note 5 (Supplementary Fig. 3).

Orbital contributions were calculated for both Sn and S at select energies and momenta in the conduction band region, and integrated over energy ranges corresponding to A_1,2_ and B_1,2_ identified in XAS. Both regions are dominated by Sn and S *s* and *p* orbitals, and contributions from Sn *d*_x,y_, *d*_y,z_, ${d_{{{\rm{z}}^2}}}$, *d*_x,z_, and ${d_{{{\rm{x}}^2} - {{\rm{y}}^2}}}$ can be neglected. A summary of the relative contributions at Γ and M is shown in Table 1. Since RPES cannot distinguish between different points in the Brillouin zone, small variations in the overall similar orbital composition at both Γ and M should be averaged (refer to Supplementary Table 2 for orbital composition at K). Regions B_1,2_ differ markedly from A_1,2_ by containing significant out-of-plane orbital character from Sn and S *p*_z_ contributions and by lacking Sn 5*s* character. As reflected in the weak spectral intensity, excitation of regions A_1,2_ is by dipole selection rules only allowed due to strong hybridization with S 3*p*_x,y_ orbitals. A_1,2_ is thus primarily composed of strongly hybridized in-plane molecular orbitals. B_1,2_ contains primarily Sn and S *p*_x,y_ and *p*_z_ character. Of these, the Sn and S *p*_z_ orbitals are more strongly hybridized due to the atomic arrangement in the unit cell (see insert Fig. 1), and B_1,2_ contains significant out-of-plane character. As a consequence, the extracted delocalization times in regions A_1,2_ reflect predominantly in-plane intralayer electron dynamics, while regions B_1,2_ report on out-of-plane interlayer processes. The atomic specificity of resonant photoemission together with an analysis of the orbital composition in the conduction band of SnS_2_ reveals thus dynamics of anisotropic charge flow in SnS_2_.

**Table 1:** Orbital compositions of SnS_2_ conduction band

		*s* (%)		*p*_x,y_ (%)		*p*_z_ (%)	
		Sn	S	Sn	S	Sn	S
Γ	A_1,2_	48.0	0.26	0	47.5	0	1.55
	B_1,2_	0.23	5.55	32.7	29.2	16.6	15.6
Μ	A_1,2_	48.1	2.31	0	42.6	0	7.08
	B_1,2_	0	11.6	13.4	17.6	31.1	26.3

Normalized orbital compositions (in %) of regions A_1,2_ and B_1,2_ at two representative high-symmetry points in the Brillouin zone. Dominant orbitals contributing include *s*, *p*_x_, *p*_y_, and *p*_z_ for both Sn and S atoms at Γ and M. Sn *d* orbitals contribute less than 3% of the total orbital contributions

**Fig. 5:**
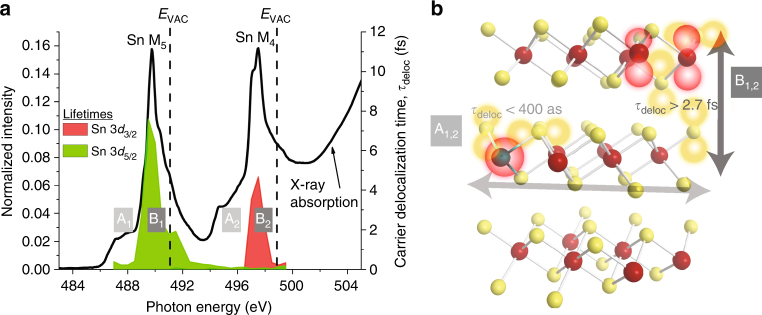
Carrier delocalization dynamics in SnS_2_. **a** Carrier delocalization times extracted from resonant photoemission spectroscopy, superimposed with XAS. Regions A_1,2_ correspond to highly delocalized conduction band electrons, while electrons excited to regions B_1,2_, deeper in the conduction band are strongly localized. **b** Cartoon of strong intralayer coupling leading to delocalization times less than 400 as, and weak interlayer coupling resulting in delocalization times greater than 2.7 fs

From Eq. (1) and the energy-dependent normal and spectator Auger intensities in Fig. 3c, we calculate the energy-dependent electron delocalization times for different regions in the conduction band. The low-resonant enhancement in regions A_1,2_ corresponds to highly delocalized charge carriers with ultrafast carrier delocalization times of ${\tau _{{\rm{deloc}},{{\rm{A}}_1}}} = \frac{1}{{{k_{{\rm{deloc}},{{\rm{A}}_1}}}}} = 376_{ - 100}^{ + 100}$ as and ${\tau _{{\rm{deloc}},{{\rm{A}}_2}}} = \frac{1}{{{k_{{\rm{deloc}},\,{{\rm{A}}_2}}}}} <$169 as, respectively, averaged over the indicated regions in Fig. 5a. In fact, the finite spectral signal-to-noise ratio in region A_2_ restricts the experimentally accessible temporal resolution of carrier delocalization to an upper bound. Since regions A_1,2_ are principally composed of hybridized in-plane Sn 5*s* and S 3*p*_x,y_ orbitals at both Γ and M and near the conduction band minimum, our results demonstrate attosecond intralayer delocalization dynamics as a consequence of strong hybridization within a single SnS_2_ layer (Fig. 5b).

In contrast, charge delocalization times in regions B_1,2_ are larger by at least an order of magnitude, i.e., ${\tau _{{\rm{deloc}},{{\rm{B}}_1}}} = \frac{1}{{{k_{{\rm{deloc}},{{\rm{B}}_1}}}}} = 3.80_{ - 0.99}^{ + 1.42}$ fs and ${\tau _{{\rm{deloc}},{{\rm{B}}_2}}} = \frac{1}{{{k_{{\rm{deloc}},{{\rm{B}}_2}}}}} = 2.68_{ - 0.67}^{ + 0.83}$ fs. This much-enhanced electron localization can be attributed to the difference in orbital composition between regions A and B: While Sn 5*p*_x,y_ and S 3*p*_x,y_ orbitals contribute at both Γ and M to this part of the conduction band too, only regions B_1,2_ have significant S 3*p*_z_ and Sn 5*p*_z_ orbital character. These out-of-plane orbitals are ultimately responsible for interlayer coupling and hence govern the delocalization between SnS_2_ layers. Symmetry considerations derived from the unit cell of 4H-SnS_2_ with symmetry group *P3m1* validate our orbital hybridization schemes in regions A_1,2_ and B_1,2_ (see Supplementary Note 1). The comparatively long carrier lifetimes in these out-of-plane orbitals, only found in regions B_1,2_ deeper in the conduction band, are clear evidence for weak interlayer coupling. Taken together, the strongly anisotropic nature of the electron dynamics in the conduction band region unambiguously indicate a strongly dynamical 2D nature already of bulk SnS_2_.

A comparison of these delocalization times with a simple estimate of hopping times obtained from the density functional theory (DFT) band structure calculations (Fig. 4 and Supplementary Fig. 3, *τ*_hop_ =  *ħ*/*W* with *W* the bandwidth) shows that such estimates (~300 as intralayer hopping and ≫1 fs interlayer hopping) are compatible with our experimentally determined delocalization times. Note, however, that hopping times and delocalization times are not expected to be identical due to the presence of the core hole in the RPES measurements and possible difficulties of DFT calculations to capture bandwidths correctly.

## Discussion

Near the conduction band minimum, regions A_1,2_ take advantage of strongly hybridized in-plane orbitals that lead to strong intralayer coupling. As a result, charge delocalization in SnS_2_ takes place on timescales of less than 400 as within a single layer. Deeper in the conduction band, regions B_1,2_ contain both in-plane and out-of-plane hybridized orbitals, and the electron dynamics access interlayer processes that occur on timescales at least one order of magnitude slower due to weak interlayer coupling. The underlying reason for this difference is the strongly anisotropic dynamic screening of charges in SnS_2_, as already reported on long timescales from static spectroscopy, e.g., of WS_2_^17^ and MoSe_2_^38^. The consequences are much more strongly screened Coulomb interactions and hence facile carrier delocalization within a layer. Coulombic interactions across layers are less screened and significantly longer range, bringing about slow interlayer charge transfer dynamics. This is also fully consistent with the band structure, which shows negligible dispersion along the Γ to A direction (see Supplementary Note 5).

Our measurements constitute the first observation of anisotropic carrier dynamics in van der Waals-layered materials, complementing efforts to tailor and understand the band structure of these materials, e.g., by ARPES^13–16^. While the extent to which layer decoupling varies across this class of materials is at present unknown, we anticipate that our approach enables investigations into variations in interlayer coupling in response to stacking symmetry, twist angle and formation of heterostructures, complementing steady-state spectroscopies such as ARPES and photoluminescence^39^. Our findings further strongly suggest the possibility to create long-lived interlayer excitons^40^ in few layer and even in bulk crystals, since interlayer delocalization between neighboring SnS_2_ sheets is at least a factor of 10 slower than intralayer delocalization. This process may be quite fast in the presence of an energetic gradient, as indicated by a recent report of interlayer charge transfer times of <50 fs in MoS_2_/WS_2_ heterostructures^41^. Note that this timescale is fully consistent with our measured interlayer delocalization times.

As a direct consequence of the anisotropic electron dynamics demonstrated here, different layers in bulk SnS_2_ are decoupled. Such layer decoupling observed dynamically provides real-time control over ultrafast electron flow even in bulk SnS_2_ in the device-relevant conduction band valleys. Indeed, while residual interlayer interactions exist, our data show that on ultrafast timescales bulk SnS_2_ acts primarily as a 2D material composed of single SnS_2_ sheets due to the highly delocalized intralayer charge dissipation and strong Coulombic localization across multiple layers. We suggest that these findings are more broadly applicable to other layered van der Waals materials such as MoS_2_. The existence of layer decoupling, directly demonstrated here on short timescales, may thus enable harnessing some of the unique properties of 2D materials in more readily available bulk materials, facilitating integration into new device platforms.

Interestingly, our resonant photoemission data also point to the existence of spin-dependent dynamics: The two excitation edges Sn M_5_ and M_4_ observed in XAS differ only in the spin–orbit character (*J* = 5/2 and 3/2, respectively). Carrier lifetimes differ by approximately a factor two, offering an opportunity for dynamical spin filtering on ultrafast timescales in SnS_2_^42^. Layer decoupling observed via dynamics on ultrafast timescales may therefore be useful not only for controlling electron dynamics, but also more generally for spin and quasiparticle currents in quasi-2D-layered materials.

## Methods

### SnS_2_ material synthesis and sample preparation

As reported elsewhere, the SnS_2_ single crystals of 4H polytype were grown using the Bridgman method and *n*-doped with chlorine to a carrier concentration of 2.3(1) × 10^17^ cm^−3^ ^43^. The SnS_2_ substrate was cleaved via mechanical exfoliation before introducing the sample to the ultrahigh vacuum preparation chamber. This was followed by pump down, baking for 12 h, and annealing at ~200 °C for 12 h. XPS measurements of Sn 3*d*_3/2_ and 3*d*_5/2_ features revealed no reduction of Sn^4+^ to Sn^2+^.

### Synchrotron experiments

All spectra were obtained at the SLAC National Accelerator Laboratory facility Stanford Synchrotron Radiation Lightsource (SSRL) on beamline 10–1. The SnS_2_ single crystals were mounted on a Mo foil using tungsten wires and annealed using a UHV button heater, followed by XPS analysis before starting the RPES measurements. The RPE spectra were acquired using a double-pass cylindrical mirror analyzer, mounted in the plane of the surface normal perpendicular to the incoming radiation. The incoming synchrotron radiation is linearly polarized with the electric field vector perpendicular to surface normal. XA measurements were performed in both TEY mode, using the drain current as well as channeltron detector current, and total fluorescence yield mode using a Si photodiode (IRD AXUV100) to differentiate between surface and bulk features, respectively. Multiple X-ray incidence angles (20°, 55°, and 90°) were measured for each absorption edge to establish angular dependence and ensure surface and bulk integrity. All spectra were calibrated against the incident X-ray photon flux measured on a gold grid installed upstream of the analysis chamber. RPES was collected at a pass energy of 25 eV in photemission mode, corresponding to 0.4 eV resolution, and the slits of the beamline spherical grating monochromator were set to approximately match this resolution for a total resolution of about 0.5 eV. The base vacuum was kept below 1 × 10^‒8^ Torr during the measurements, and sample integrity was checked before and after each RPES run using XPS. Photon energy calibration was achieved by fixing S 2*p*_1/2_ and S 2*p*_3/2_ at 162.5 and 163.6 eV binding energies, respectively, and by XA scans conducted before and after each RPES data set.

### Spectral corrections

A linear background subtraction followed by an integrated background subtraction was performed on all XP and XA spectra. All spectral intensities were normalized with respect to X-ray photon flux (*I*_0_) of the incoming synchrotron radiation calibrated on a gold grid upstream from the sample. Reference scans of S 2*p*_1/2_ and S 2*p*_3/2_ were measured after each RPES and XA scan for energy calibration and global vacuum-level corrections.

### Electronic structure calculations

First-principle DFT was performed using the projector-augmented wave method^44^ as implemented in the VASP package^45^. In order to account for dispersion interactions during structure optimization and electronic structure calculations, an empirical dispersion correction method proposed by Grimme et al. was used^46^. The atomic coordinates were optimized using both the non-local-correlated Perdew-Burke-Ernzerhof (PBE) functional and a hybrid functional (HSE)^47^. For the HSE calculations, Hartree–Fock screening and exchange parameters, with a PBE correlation, were set to 0.2 Å^−1^ and 25%, respectively, known as the HSE06 flavor of the available hybrid functionals within the VASP package. A zone-centered Monkhorst–Pack scheme^48^ was adopted to integrate over the Brillouin zone with a *k*-mesh of 8 × 8 × 4 for the SnS_2_ structure, and a plane-wave basis kinetic energy cutoff of 300 eV was used. To represent the crystalline solid, periodic boundary conditions in all three spatial dimensions were applied. The total energies and Hellmann–Feynman forces on the atoms were converged to 10^−6^ eV and 50 meV Å^−1^, respectively. Space and momentum-projected DOS calculations and band structure calculations were performed by integrating over the Brillouin zone with a 16 × 16 × 8 *k*-mesh using the tetrahedron method with Bloch corrections^44^. Spin–orbit coupling was included during density of states (DOS) and band structure calculations.

### Data availability

The data that support the findings of this study are available from the corresponding author on request.

## Electronic supplementary material

Supplementary Information

Peer Review File

## References

[1] Jin W (2013). Direct measurement of the thickness-dependent electronic band structure of MoS_2_ using angle-resolved photoemission spectroscopy. Phys. Rev. Lett..

[2] Mak KF, Lee C, Hone J, Shan J, Heinz TF (2010). Atomically thin MoS_2_: a new direct-gap semiconductor. Phys. Rev. Lett..

[3] Zhang Y (2014). Direct observation of the transition from indirect to direct bandgap in atomically thin epitaxial MoSe_2_. Nat. Nanotechnol..

[4] Gong Z (2013). Magnetoelectric effects and valley-controlled spin quantum gates in transition metal dichalcogenide bilayers. Nat. Commun..

[5] Riley JM (2015). Negative electronic compressibility and tunable spin splitting in WSe_2_. Nat. Nanotechnol..

[6] Yuan H (2016). Evolution of the valley position in bulk transition-metal chalcogenides and their monolayer limit. Nano Lett..

[7] Mai C (2014). Many-body effects in valleytronics: direct measurement of valley lifetimes in single-layer MoS_2_. Nano Lett..

[8] Sallen G (2012). Robust optical emission polarization in MoS_2_ monolayers through selective valley excitation. Phys. Rev. B.

[9] Georgiou T (2013). Vertical field-effect transistor based on graphene-WS_2_ heterostructures for flexible and transparent electronics. Nat. Nanotechnol..

[10] Desai SB (2016). MoS_2_ transistors with 1-nanometer gate lengths. Science.

[11] Bernardi M, Palummo M, Grossman JC (2013). Extraordinary sunlight absorption and one nanometer thick photovoltaics using two-dimensional monolayer materials. Nano Lett..

[12] Okamoto N (2014). Electric control of the spin Hall effect by intervalley transitions. Nat. Mater..

[13] Alidoust N (2014). Observation of monolayer valence band spin-orbit effect and induced quantum well states in MoX_2_. Nat. Commun..

[14] Jin W (2013). Direct measurement of the thickness-dependent electronic band structure of MoS_2_ using angle-resolved photoemission spectroscopy. Phys. Rev. Lett..

[15] Miwa JA (2015). Electronic structure of epitaxial single-layer MoS_2_. Phys. Rev. Lett..

[16] Yeh P-C (2016). Direct measurement of the tunable electronic structure of bilayer MoS_2_ by interlayer twist. Nano Lett..

[17] Chernikov A (2014). Exciton binding energy and nonhydrogenic Rydberg series in monolayer WS_2_. Phys. Rev. Lett..

[18] Racke DA, Neupane MR, Monti OLA (2016). Valence and conduction band structure of the quasi-two-dimensional semiconductor SnS_2_. Phys. Rev. B.

[19] Tongay S (2014). Monolayer behaviour in bulk ReS_2_ due to electronic and vibrational decoupling. Nat. Commun..

[20] Riley JM (2014). Direct observation of spin-polarized bulk bands in an inversion-symmetric semiconductor. Nat. Phys..

[21] Gehlmann M (2016). Quasi 2D electronic states with high spin-polarization in centrosymmetric MoS_2_ bulk crystals. Sci. Rep..

[22] Reed JP (2010). The effective fine-structure constant of freestanding graphene measured in graphite. Science.

[23] Huang Y (2014). Tin disulfide—an emerging layered metal dichalcogenide semiconductor: materials properties and device characteristics. ACS Nano.

[24] Racke DA, Monti OLA (2014). Persistent non-equilibrium interface dipoles at quasi-2D organic/inorganic semiconductor interfaces: the effect of gap states. Surf. Sci..

[25] Racke DA, Kelly LL, Monti OLA (2015). The importance of gap states for energy level alignment at hybrid interfaces. J. Electron Spectrosc. Relat. Phenom..

[26] Brühwiler P, Karis O, Mårtensson N (2002). Charge-transfer dynamics studied using resonant core spectroscopies. Rev. Mod. Phys..

[27] Kumar N, He J, He D, Wang Y, Zhao H (2013). Charge carrier dynamics in bulk MoS_2_ crystal studied by transient absorption microscopy. J. Appl. Phys..

[28] Kumar N (2014). Exciton diffusion in monolayer and bulk MoSe_2_. Nanoscale.

[29] Bertoni R (2016). Generation and evolution of spin-, valley-, and layer-polarized excited carriers in inversion-symmetric WSe_2_. Phys. Rev. Lett..

[30] Wallauer R, Reimann J, Armbrust N, Güdde J, Höfer U (2016). Intervalley scattering in MoS_2_ imaged by two-photon photoemission with a high-harmonic probe. Appl. Phys. Lett..

[31] Xu S (1997). Ultrafast electron dynamics in two dimensional layered systems: two-photon photoemission studies of SnS_2_. Chem. Phys. Lett..

[32] Föhlisch A (2005). Direct observation of electron dynamics in the attosecond domain. Nature.

[33] Menzel D (2008). Ultrafast charge transfer at surfaces accessed by core electron spectroscopies. Chem. Soc. Rev..

[34] Parry-Jones AC, Weightman P, Andrews PT (1979). The M_4,5_N_4,5_N_4,5_ Auger spectra of Ag, Cd, In and Sn. J. Phys. C.

[35] Mårtensson N, Nyholm R (1981). Electron spectroscopic determinations of M and N core-hole lifetimes for the elements Nb - Te (Z = 41-52). Phys. Rev. B.

[36] Campbell JL, Papp T (1995). Atomic level widths for X-ray spectrometry. X-Ray Spectrom..

[37] McGuire EJ (1972). Atomic M-shell Coster-Kronig, Auger, and radiative rates, and fluorescence yields for Ca-Th. Phys. Rev. A.

[38] Ugeda MM (2014). Giant bandgap renormalization and excitonic effects in a monolayer transition metal dichalcogenide semiconductor. Nat. Mater..

[39] Xia J, Yan J, Shen ZX (2017). Transition metal dichalcogenides: structural, optical and electronic property tuning via thickness and stacking. FlatChem.

[40] Rivera P (2015). Observation of long-lived interlayer excitons in monolayer MoSe_2_–WSe_2_ heterostructures. Nat. Commun..

[41] Hong X (2014). Ultrafast charge transfer in atomically thin MoS_2_/WS_2_ heterostructures. Nat. Nanotechnol..

[42] Steil S (2013). Spin-dependent trapping of electrons at spinterfaces. Nat. Phys..

[43] Sharp L, Soltz D, Parkinson BA (2006). Growth and characterization of tin disulfide single crystals. Cryst. Growth Des..

[44] Blöchl PE (1994). Projector augmented-wave method. Phys. Rev. B.

[45] Kresse G, Furthmüller J (1996). Efficient iterative schemes for ab initio total-energy calculations using a plane-wave basis set. Phys. Rev. B.

[46] Grimme S, Antony J, Ehrlich S, Krieg H (2010). A consistent and accurateab initio parametrization of density functional dispersion correction (DFT-D) for the 94 elements H-Pu. J. Chem. Phys..

[47] Heyd J, Scuseria GE (2003). Hybrid functionals based on a screened Coulomb potential. J. Chem. Phys..

[48] Monkhorst HJ, Pack JD (1976). Special points for Brillouin-zone integrations. Phys. Rev. B.

