# Supplementary material for: Anisotropic attosecond charge carrier dynamics and layer decoupling in quasi-2D layered SnS2
Source: Nat Commun. 2017 Nov 8;8:1369. doi: 10.1038/s41467-017-01522-3 (PMC5678129; doi:10.1038/s41467-017-01522-3)
Supplement: Supplementary file 1 — Supplementary Information [file 41467_2017_1522_MOESM1_ESM.pdf]

Supplementary Note 1. Symmetry considerations of orbital hybridization. To establish possible hybridization schemes of the atomic orbitals that constitute the SnS_2 conduction band, we create symmetry-adapted linear combinations of atomic orbitals. Irreducible representations of the relevant atomic orbitals, s, $p_{\mathrm{x,y}}$ and p_{z} on both Sn and S atoms are obtained in the C_{3v} point group, since 4H-SnS_2 belongs to the *P3m1* space group. Orbitals for atoms located on the C_3 axis of the unit cell transform according to the following irreducible representations: $s \rightarrow A_1$, $p_{\mathrm{x,y}} \rightarrow E$, $p_{\mathrm{z}} \rightarrow A_1$. Similarly, atoms located on the corners and edges of the unit cell transform as $s \rightarrow A_1 + E$, $p_{\mathrm{x,y}} \rightarrow A_1 + A_2 + 2E$, $p_{\mathrm{z}} \rightarrow A_1 + E$. With these irreducible representations we construct the symmetry-allowed hybridization of Sn and S orbitals as designated in the main text and delineated in Supplementary Table 2: Sn 5s and S 3$p_{\mathrm{x,y}}$ each transform as A_1, and regions $A_{1,2}$ in the XA-spectrum represent bands with a hybridized Sn 5s $\otimes$ S 3$p_{\mathrm{x,y}}$ character. In regions $B_{1,2}$, Sn 5$p_{\mathrm{x,y}}$ and S 3$p_{\mathrm{x,y}}$ each contain E character, while Sn 5p_{z} and S 3p_{z} both transform as A_1. Thus, the conduction band in regions $B_{1,2}$ is dominated by two hybridized bands, Sn 5$p_{\mathrm{x,y}}$ $\otimes$ S 3$p_{\mathrm{x,y}}$ and Sn 5p_{z} $\otimes$ 3p_{z}. Given the selection rules and excitation geometry, this establishes that region A represents predominantly intralayer electronic states, while region B contains interlayer out-of-plane orbital character.

Supplementary Note 2. Surface defects not detected in the conduction band of SnS_2. To consider possibly important contributions from surface defects[1,2], we compare the surface-sensitive TEY (total electron yield) XA spectrum with bulk-sensitive TFY (total fluorescence yield) XA spectrum in Supplementary Fig. 1. Despite the lower signal-to-noise ratio typical for TFY XA, both spectral features within each spin-orbit component are clearly present, indicating that surface defect states play at most a minor role in the core-level spectroscopy. This is

important for loss mechanisms following excitation to the conduction band such as charge recombination or localization effects, which would likely hinder charge delocalization within the 2D layered material of SnS_2 by defect scattering. Our results show that such effects can be neglected.

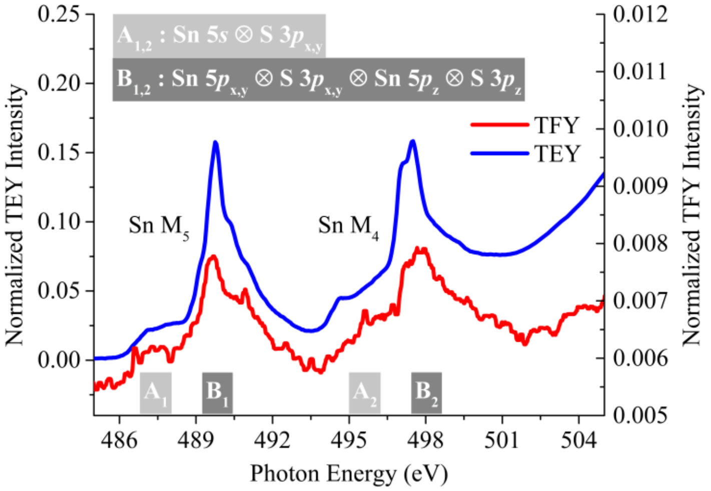

Supplementary Figure 1. Conduction band of SnS_2. X-ray absorption spectra of SnS_2 on Sn $M_{4,5}$ edges, with calculated principal orbital composition indicated. Surface-sensitive TEY and bulk sensitive TFY shows the same features, indicating that XAS is probing the bulk electronic structure of SnS_2 across multiple layers.

Supplementary Note 3. Normal and spectator Auger fits. In order to isolate the spectator Auger contribution in the RPES map, we first analyze the non-resonant or normal Auger component, which is largely independent of photon energy and detected on the M_5 edge at photon energies below 488 eV and above 510 eV. The dominant Auger channel Sn $M_{4,5}N_{4,5}N_{4,5}$ corresponds to the Sn $3d^{10}4d^{10} \rightarrow$ Sn $3d^{10}4d^{9} + \mathrm{e}^{-} \rightarrow$ Sn $3d^{10}4d^{8} + \mathrm{e}^{-}$ transition. Supplementary Figure 2

illustrates the normal Auger components for both Sn M_4 and Sn M_5 edges off-resonance at a photon energy of 520 eV.

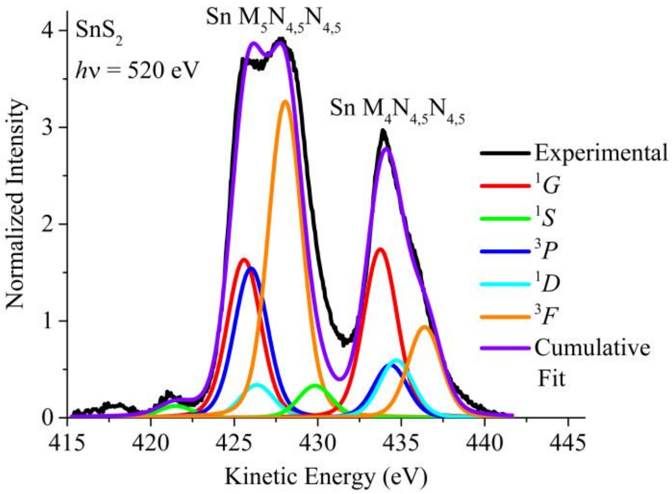

Supplementary Figure 2. General fit for normal and spectator Auger features. Fit of normal Auger spectra of Sn $M_4N_{4,5}N_{4,5}$ and Sn $M_5N_{4,5}N_{4,5}$ at a photon energy of 520 eV with explicitly shown atomic final states of ^1G, ^1S, ^3P, ^1D and ^3F. The cumulative fit shows excellent agreement with respect to the experimental curve.

The associated spectral envelope can be fit by decomposition into the final state atomic states, considering selection rules and transition matrix elements between a d^{10} initial state and a d^8 two-hole final state. Five atomic term symbols ^1S, ^1G, ^3P, ^1D and ^3F describe the accessible final states in both Sn $M_{4,5}N_{4,5}N_{4,5}$ Auger transitions. The Auger kinetic energy for each of these atomic terms (X) can be decomposed as follows:[3]

$$KE(\mathrm{M}_{4,5}\mathrm{N}_{4,5}\mathrm{N}_{4,5};X) = BE(\mathrm{M}_{4,5}) - BE(\mathrm{N}_{4,5}) - BE(\mathrm{N}_{4,5}) - F(\mathrm{N}_{4,5}\mathrm{N}_{4,5};X) + B(\mathrm{N}_{4,5}\mathrm{N}_{4,5};X) \quad (1)$$

F (Coulomb term) is expanded in a multi-pole expansion up to the 4th order resulting in three integrals to be fit, and B (relaxation energy) compensates for screening of the core-excited electron. Both spin-orbit components were fit simultaneously using four energy parameters (three F integrals and one B term), peak intensities and linewidths. The floating energy parameters describe the resulting kinetic energy positions of the atomic features and the spectral offset relative to the ^1G term. While some intensities could be floated, ^1G and ^3P intensities needed to be fixed due to strong overlap in the congested spectra. Each feature was fit using a Voigt peak profile of 1:5 Lorentzian:Gaussian peak character to take into account spectral broadening effects. Despite being composed of many atomic transitions, the spectra could in fact be fit with only a minimal number of parameters. Importantly, except for photon energy-dependent intensities, all other parameters were robust in the global fitting scheme.

Supplementary Note 4. Comparison of experimental SnS_2 normal Auger fit to theoretical Sn Auger fit. Fit results are presented in Supplementary Table 1 and compared with theoretical calculations of metallic Sn[4], showing generally excellent agreement. Some differences arise, primarily on the M_4 edge, likely due to an oversimplification of the theoretical treatment of metallic Sn which uses a free-atom model that does not account for solid-state and instrumental effects.

Eigenvector	ΔKinetic Energy $M_{4,5}N_{4,5}N_{4,5}$ (eV)		Intensity, $M_5N_{4,5}N_{4,5}$		Intensity, $M_4N_{4,5}N_{4,5}$	
	Sn, theory	SnS_2, exp	Sn, theory	SnS_2, exp	Sn, theory	SnS_2, exp
^1S	-4.14	-3.86 +/- 0.09	0.13	0.07 +/- 0.04	0.25	0.20 +/- 0.37
^1G	0	0 +/- 0.13	1.00	1.00 +/- 0.05	1.16	1.06 +/- 0.04
^3P	0.55 - 1.28$^\zeta$	0.57 +/- 0.07	0.78	0.94 +/- 0.05	0.85	0.33 +/- 0.04
^1D	0.91	0.97 +/- 0.03	0.20	0.21 +/- 0.05	0.93	0.36 +/- 0.04
^3F	2.25 - 3.36$^\zeta$	2.65 +/- 0.01	2.02	2.00 +/- 0.37	0.94	0.57 +/- 0.04

Supplementary Table 1. Experimental fit parameters compared to theory. Comparison of experimentally determined and theoretical calculated kinetic energies and intensities of Sn $M_{4,5}N_{4,5}N_{4,5}$ of d^8 final states in SnS_2 and metallic Sn, respectively. $^\zeta$Contains two spin-orbit components not resolved in the experiment.

Supplementary Note 5. Orbital composition at all high symmetry points and out-of-plane band structure. High-level electronic structure calculations at Γ, M and K points provide similar orbital contributions in regions $A_{1,2}$ and $B_{1,2}$ as delineated in Table 1 of the main text. Supplementary Table 2 gives additional s, p_x, p_y and p_z orbital contributions at K not specified in Table 1. Supplementary Figure 3 provides an out-of-plane band structure diagram showing the flat bands in the valence and conduction band regions further demonstrating a lack of dispersion compared to in-plane band structure (Fig. 4).

		s (%)		$p_{x,y}$ (%)		p_z (%)	
		Sn	S	Sn	S	Sn	S
Γ	$A_{1,2}$	48.0	0.26	0	47.5	0	1.55
	$B_{1,2}$	0.23	5.55	32.7	29.2	16.6	15.6
M	$A_{1,2}$	48.1	2.31	0	42.6	0	7.08
	$B_{1,2}$	0	11.6	13.4	17.6	31.1	26.3
K	$A_{1,2}$	49.3	0	0	50.5	0	0.26
	$B_{1,2}$	0	8.10	48.6	17.3	0.75	25.2

Supplementary Table 2. Orbital composition of conduction band at all high-symmetry points. Normalized orbital compositions of regions $A_{1,2}$ and $B_{1,2}$ with respect to all high-symmetry points in the Brillouin zone, Γ, M and K. Sn and S atoms have the following major orbital contributions: s, p_x, p_y and p_z.

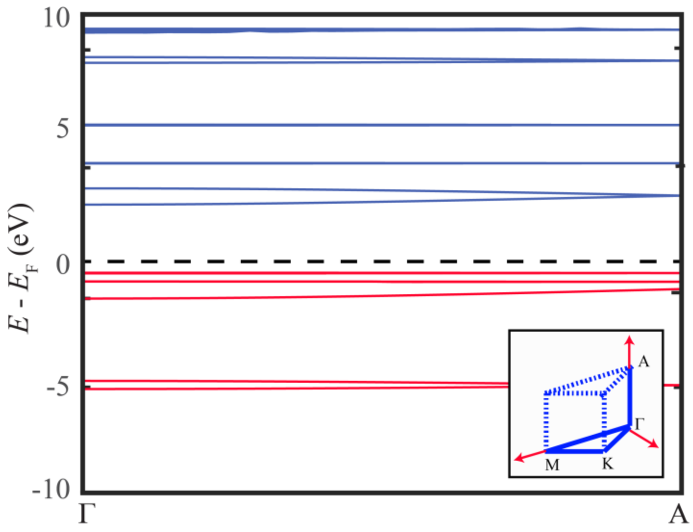

Supplementary Figure 3. Out-of-plane band structure of SnS_2. Electronic band structure calculation at high-symmetry points Γ to A.

Supplementary References

1. Kucheyev, S. O. *et al.* Surface electronic states in three-dimensional SnO_2 nanostructures. *Phys. Rev. B* **72,** 035404 (2005).

2. Chuvenkova, O. A. *et al.* XANES and XPS investigations of surface defects in wire-like SnO_2 crystals. *Phys. Solid State* **57,** 153–161 (2015).

3. Slater, J. C. *Quantum Theory of Atomic Structure*. **II,** (New York, McGraw-Hill, 1960).

4. Parry-Jones, A. C., Weightman, P. & Andrews, P. T. The $M_{4,5}N_{4,5}N_{4,5}$ Auger spectra of Ag, Cd, In and Sn. *J. Phys. C Solid State Phys.* **12,** 1587 (1979).
